# Anthropogenic influence on extreme temperature and precipitation in Central Asia

**DOI:** 10.1038/s41598-023-33921-6

**Published:** 2023-04-26

**Authors:** Bijan Fallah, Emmanuele Russo, Christoph Menz, Peter Hoffmann, Iulii Didovets, Fred F. Hattermann

**Affiliations:** 1 Potsdam Institute for Climate Impact Research (PIK), Telegrafenberg A62, 14473 Potsdam, Brandenburg Germany; 2 Institute for atmospheric and climate science, ETH Zürich, Universitätstrasse 16, 8092 Zürich, Switzerland

**Keywords:** Climate change, Attribution

## Abstract

We investigate the contribution of anthropogenic forcing to the extreme temperature and precipitation events in Central Asia (CA) during the last 60 years. We bias-adjust and downscale two Inter-Sectoral Impact Model Intercomparison Project (ISIMIP) ensemble outputs, with natural (labelled as *hist-nat*, driven only by solar and volcanic forcing) and natural plus anthropogenic forcing (labelled as *hist*, driven by all-forcings), to $0.25^{\circ } \times 0.25 ^{\circ }$ spatial resolution. Each ensemble contains six models from ISIMIP, based on the Coupled Model Inter-comparison Project phase 6 (CMIP6). The presented downscaling methodology is necessary to create a reliable climate state for regional climate impact studies. Our analysis shows a higher risk of extreme heat events (factor 4 in signal-to-noise ratio) over large parts of CA due to anthropogenic influence. Furthermore, a higher likelihood of extreme precipitation over CA, especially over Kyrgyzstan and Tajikistan, can be attributed to anthropogenic forcing (over 100$\%$ changes in intensity and 20$\%$ in frequency). Given that these regions show a high risk of rainfall-triggered landslides and floods during historical times, we report that human-induced climate warming can contribute to extreme precipitation events over vulnerable areas of CA. Our high-resolution data set can be used in impact studies focusing on the attribution of extreme events in CA and is freely available to the scientific community.

## Introduction

**Figure 1:**
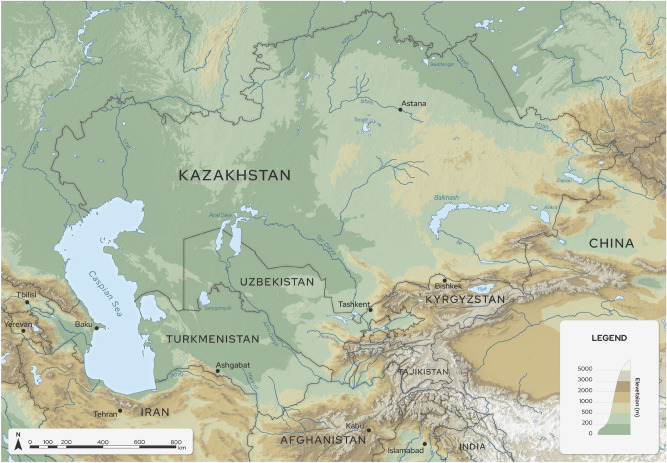
Central Asian domain and topography(m). The boundary data were taken from Natural Earth. Free vector and raster map data at www.naturalearthdata.com. The basin and river data were taken from HydroATLAS^34^. The elevation data were downloaded from the ASTER Global Digital Elevation Map^35^. The map was created using ArcGIS V10.8.2 (https://www.esri.com), Adobe Illustrator Version 27.2 (www.adobe.com) and Blender V3.4.1 (www.blender.org).

Water availability, hydro-power, and food security are the main concerns for Central Asian (CA) society in the Anthropocene^1^. Although the 1.5-degree target has not been proven impossible to achieve, many future climate scenarios project global warming to surpass this critical level at the end of the century compared to pre-industrial^2^. The societal and economic impacts of global warming will be severe if this threshold is reached^1^. At the regional scale, CA’s recent positive temperature trend is already significantly above the global mean^3^. Also, the precipitation amount, snow-melt/glacier ratio, and precipitation phase (rain/snow) have been modified in recent decades^4^. The snow-to-precipitation ratio has shown a negative trend in recent decades. For example, more than $97\%$ of the Tian Shan snow cover has started to retreat since the 1980s^4^. It is well-known that global warming will affect the hydrological cycle based on the Clausius-Clapeyron relation of thermodynamic theory and changes in the atmospheric circulation, increasing its intensity and frequency^5^. The observed recent warming increased the frequency of daily precipitation extremes by $18\%$ over land compared to pre-industrial^6^. Rain collected over Tian Shan affects the five CA countries selected in this study (Fig. 1): Tajikistan and Kyrgyzstan are the upstream, and Turkmenistan, Uzbekistan, and Kazakhstan are the downstream countries of rivers’ flow. Kyrgyzstan and Tajikistan are countries exposed to extreme landslide hazards and extreme precipitation. The complex political interrelations (i.e. the collapse of the Soviet Union) and the climate change impacts make it challenging to tackle the water availability problem in CA. Increasing extreme precipitation and warming temperature can contribute to more severe floods/droughts and glacier melting, with a series of devastating implications on the economy of these regions (e.g., malfunctioning in the transport infrastructure), as well as health and food security^7^. More frequent heat waves^8^ and the expansion of arid areas in CA^9^ are likely to reduce crop productivity. With 4$^\circ \textrm{C}$ of warming in CA to pre-industrial times, there will most probably be decision-making conflicts between the different countries of the area. For example, the challenge would be whether to use the water solely for agriculture or hydro-power generation^1^. The complexity of the climate system makes it challenging to distinguish between naturally forced and human-induced extreme events^10^, especially at the regional scale. It is hard to distinguish the climate’s response to anthropogenic influence from the natural internal and externally forced (solar or volcanic forcings) climate variability^11^. For example, the Russian heat wave of 2010 can be linked to internal variability in the magnitude and driven by external forcing in terms of its occurrence probability^12^. In the detection process, one must demonstrate that the observed changes are significantly different from a situation explained by natural internal variability^13^. To attribute those changes to human activity, one has to demonstrate that the observed changes are (1) unlikely related only to internal variability, (2) consistent with the responses to the anthropogenic and natural forcing, and (3) there exists no physical explanations of recent climate change without the combination of natural and anthropogenic forcings^11^.

Many studies have explored the impact of global warming on weather extremes in general circulation models (GCMs), i.e., extreme sea level rise^14^, rare rainfall events^15^, hydro-climatic extremes^16^, tropical cyclones^17^ and heatwaves^18^. However, the high-resolution spatiotemporal pattern of climate change is less discussed^19^, particularly robust attribution of extreme precipitation events over CA. Detection and attribution are challenging at the regional scale due to a small signal-to-noise ratio^19^. Furthermore, due to the internal variability, the observed and simulated responses to anthropogenic forcing contain sampling uncertainty^20^. A classical approach to extreme event attribution is to run a large ensemble of coupled climate simulations with and without anthropogenic forcing during the historical era (i.e., 1850–2014) and compare the statistics of the two model sets. According to the central limit theorem, this will reduce the sampling uncertainty but could only eliminate it if the number of simulations *N* reaches the $\infty$. Models are not expected to reconstruct precisely the observed evolution of the chaotic internal variability but should capture the statistics of the climate system’s variability (“noise”)^21^. Models are assumed to capture the response to the external forcing correctly if the ones driven by human and natural forcing are consistent with the observed changes and those without human influence are inconsistent. However, most attribution studies assume that models correctly capture the shape of the response to external forcings (the large-scale spatiotemporal patterns). Therefore the magnitude of the response shall not be simulated accurately.

Generally, the attribution of temperature extremes shows a robust signal due to climate regime shifts in the data: the probability distribution of temperature anomalies is usually shifted towards warmer values. Changes in temperature distributions might be on the mean, variability, shape, or a mixture of all^22^. However, extreme precipitation events are poorly simulated by the models, identified by the observations, and do not follow a general trend^23^. This is partially due to insufficient parameterization in GCMs, e.g., parameterization of small-scale but highly influential processes like convective precipitations. There exists larger variability for simulated precipitation among different models. This originates from the large uncertainties in the observations^24^, different boundary data, the unforced “internal variability” within models, oversimplification of the models and numerical implementation, etc^25^. The impact of human activity on the probability of extreme events like floods, droughts, and storms is somewhat complex and mixed^26^. Even for a very dense Alpine observation network, the precipitation pattern and magnitude are critically dependent upon the analysis and observation density. In CA, the number of station data with daily values is very small, and the spatial gridded data sets have higher confidence in monthly values. Climate impact assessments at regional and national scales require high-resolution climate data. GCM outputs are usually downscaled at higher resolutions for climate impact modelling like hydrological simulations^27^, adaptation strategies^28^, and agriculture studies^29^. Additionally, impact models are usually tuned by local observations. Therefore, climate model outputs shall be bias-adjusted for climate impact modelling. To improve the problems of low resolution, mixed anthropogenic and natural forcing and the model uncertainties, we apply bias adjustment and statistical downscaling over an ensemble of climate model simulations to make their statistics, more similar to high-resolution observational data^30,31^. The ISIMIP project aims to model the impacts of climate change comprehensively. In the counterfactual ISIMIP ensemble, two sets of model simulations from CMIP6 with and without anthropogenic forcings labelled as “historical” and “hist-nat”, are bias-adjusted using a gridded observational dataset and statistically downscaled to a $0.5^\circ \times 0.5^\circ$ horizontal resolution. This resolution is appropriate for impact studies over river basins larger than 50,000 $km^{2}$^32^. For impact studies at smaller regional scales in CA, a higher resolution is required to have enough grid points of the model within those basins. Using the ISIMIP data, impact modellers can contribute to obtaining a comprehensive picture of the world under different climate change scenarios and disentangle the anthropogenic and natural forcing. The fifth generation CMIP5 model outputs have been shown to underestimate extreme precipitations globally^33^.

This paper explores the anthropogenic impact of CA’s extreme temperature and precipitation events from 1961 to 2014. First, we explain the methodology used for creating the climate data. Furthermore, we discover the contribution of anthropogenic forcing to changes in the frequency and magnitude of extreme temperature events and their spatial patterns. Then we analyze the statistics of precipitation extremes to study the impact of anthropogenic forcing. Finally, we discuss the correlation between the intensified precipitation extremes and the occurrence of land-slide events in CA.

## Data and methods

We use a high-resolution gridded observational data set and a bias adjustment/statistical downscaling approach to increase the resolution of two sets of CMIP6-based ISIMIP products, i.e., *hist* (driven by natural and anthropogenic forcings) and *hist-nat* (driven only by natural forcing). To our knowledge, no study has used high-resolution bias-adjusted CMIP6 products to explore the influence of anthropogenic forcing on the extreme precipitation and temperature in CA. It has been previously shown^31^ that this methodology creates a robust climate state in which the climate trends from CMIP6 are preserved in the statistics of the higher-resolution dataset. Considering the computational expenses of dynamical downscaling approaches, the presented methodology is, in our opinion, the best alternative in the impact modelling community. In this study, we use the Climatologies at High resolution for the Earth’s Land Surface Areas (CHELSA^36^) data set as our high-resolution observations (1 km), which is based on the statistical downscaling of a new generation of reanalysis data, the European Centre for Medium-Range Weather Forecasts (ECMWF) ERA5^37,38^. We bias-adjust and downscale a set of previously bias-adjusted and statistical downscaled data sets of phase 3 of ISIMIP (ISIMIP3b^39^) to a $0.25^{\circ } \times 0.25 ^{\circ }$ horizontal resolution. These simulations were conducted with natural plus anthropogenic (hereafter, *hist*) and with natural only (hereafter, *hist-nat*) forcing from 1961 to 2014. The downscaled fields show a smoother pattern in an approach using multiple steps than using one big step^30^. We have chosen the final $0.25^{\circ } \times 0.25 ^{\circ }$ horizontal resolution as a trade-off between computational expenses, model performance, and the expectations from climate impact modellers^40^. With the coarse observational network in CA, the spatiotemporal statistics of the original 1km data can not be evaluated against the station data. In the following, we present our analysis of extreme temperature and precipitation in CA. Table 1 summarises the gridded datasets used in this study.

**Table 1:** Gridded data sets used in this study.

Data	Time range (step)	Resolution	Usage	Variables
CHELSA^36^	1979–2016 (daily)	1km$\times$1km	Bias-adjustment and statistical downscaling	Total precipitation/near-surface temperature
NOAA-CIRES-DOE 20th Century Reanalysis version 3 ensemble mean^41^	1836–2015 (3−hourly)	$1^{\circ } \times 1^{\circ }$	Evaluation	Total precipitation/near-surface temperature
Berkeley-Earth^42^	1880–recent (daily)	$1^{\circ } \times 1^{\circ }$	Evaluation	Near-surface temperature
ISIMIP3b bias-adjusted atmospheric climate input data^43^	1850–2020 (daily)	$0.5^{\circ } \times 0.5^{\circ }$	Input climate data	Total precipitation/near-surface temperature

**Figure 2:**
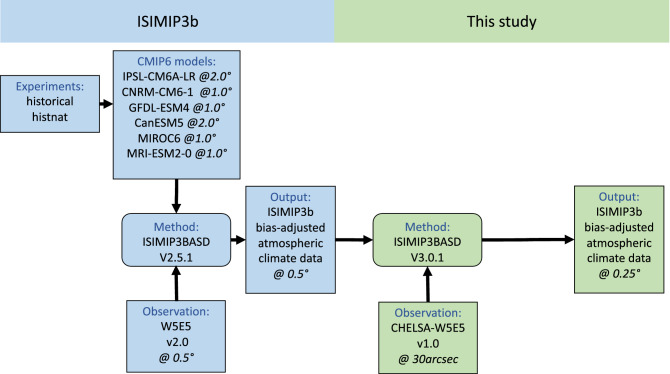
Schematic of the ISIMIP3b (blue) and this study’s approach (green). Boxes with sharp (rounded) corners represent data (algorithms). The spatial resolution of different data sets is indicated in italics.

Figure 2 shows the schematic of the processes used to create the climate output data in ISIMIP3b and this study. The bias adjustment in ISIMIP3b is based on a parametric quantile mapping method which reduces the error in all distribution quantiles and preserves the trends in each of them^31^. The new version V3.0.1 improved the distribution fits for parametric quantile mapping and therefore outperforms its predecessors^31^. For a complete explanation of the BASD method, we refer to the ISIMIP3b fact sheet available via https://www.isimip.org/documents/413/ISIMIP3b_bias_adjustment_fact_sheet_Gnsz7CO.pdf and the references therein^30,31,43^.

By combining the ERA5 precipitation and MODIS satellite monthly cloud cover frequency, CHELSA precipitation improved spatial and temporal accuracy in complex terrain compared to the existing gridded data sets^38^. The ISIMIP3b data is bias-adjusted using the W5E5 v2.0^30^. The spatially aggregated CHELSA and W5E5 v2.0 show similar statistics for precipitation and temperature like mean, median and PR98^36^. However, before downscaling the ISIMIP3b data set, we bias-adjust it again using the aggregated CHELSA at 0.5$^{\circ }$ (suggestion of the code developer, Stefan Lange). Changes introduced by the additional BA can be considered minor due to the similarity of CHELSA and W5E5. The aggregation of the CHELSA to the climate grid was done using first-order conservative remapping^44^. For creating the composite pattern of precipitation during the landslide events, we consider the 3-day running mean values. For estimation of the temperature probability density functions (PDFs), we use the kernel density using Gaussian kernels^45^ implemented in the scipy (https://docs.scipy.org/doc/scipy/reference/generated/scipy.stats.gaussian_kde.html, last visited on 25.01.2023) library of python. This method has been used in several related studies and has shown to be robust^46^. Following the ISIMIP3b protocol^43^, we first remove the biases of the model output (for the list of the considered models, see the Data and Methods section) using CHELSA. Then, we statistically downscale the output (from 0.5$^{\circ } \times 0.5^{\circ }$ to 0.25$^{\circ } \times 0.25^{\circ }$) using a stochastic approach (see “Data and methods” and Fig. 2). It is essential to conduct statistical downscaling in multiple small steps (like in this study) instead of one big step (from the CMIP6 original resolution of 1 or 2$^{\circ } \times 1$ or 2° to 0.25$^{\circ } \times 0.25 ^{\circ }$ horizontal resolution) as shown previously by statistical downscaling using neural networks^47^. The new ISIMIP3b statistical downscaling software is spatially more coherent than simple uni-variate methods. It uses a stochastic approach to avoid mismatches between the coarse-resolution model, and finer observational data sets^48^. The algorithm applies a uni-variate empirical quantile mapping using multiple grid boxes of the dataset^30^. The downscaling method^49^ preserves the coarse-scale statistics (like the trends). The ISIMIP3b bias-adjustment method is univariate and parametric.

**Figure 3:**
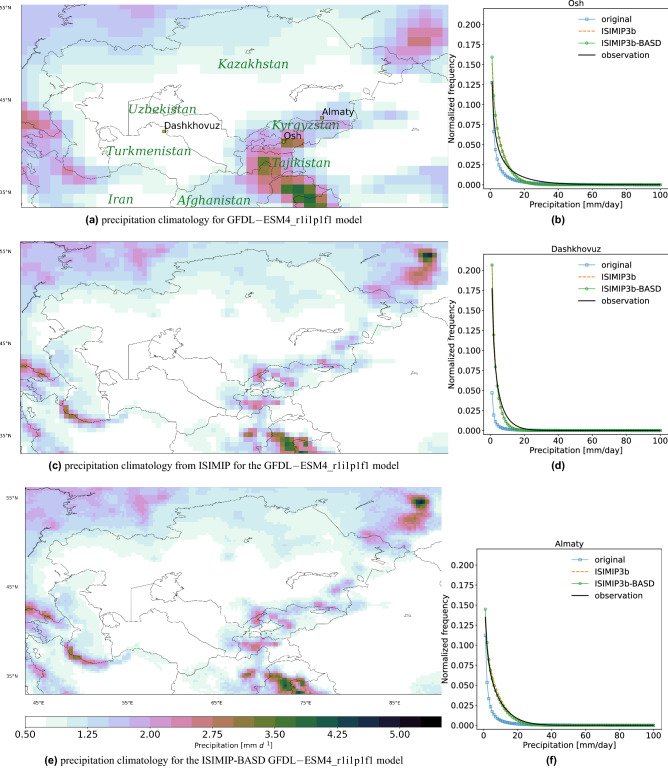
Climatology of precipitation [mm/day] for (**a**) GFDL−ESM4$\_$r1i1p1f1 model original grid ($\Delta$x=1.25$^{\circ }$ and $\Delta$y=1$^{\circ }$), (**c**) ISIMIP3b at 0.5$^{\circ }$ and (**e**) this study at 0.25 $^{\circ }$. The precipitation fields show the average for 1985-2014 over Central Asia. Model-data comparison of precipitation frequencies for three available observations, shown in (**a**) for 1985–2000 (**b**,**d**,**f**). The maps were created using python3-matplotlib (version 3.1.2, https://matplotlib.org/).

For a local impact study at country or river basin scales, especially for regions such as Kyrgyzstan or Tajikistan, characterized by very complex topography, scientists require high-resolution (less than 30 km) climate data^49^. Figure 3a,c,e show the different grid structures of the climatological precipitation pattern over the CA domain as calculated from one of the original CMIP6 GCMS (only GFDL-ESM4 is shown here), its corresponding ISIMIP3b and additional bias-adjusted and statistically downscaled ISIMIP3b (ISIMIP3b-BASD, hereafter) from top to bottom, respectively. Although a more refined precipitation structure is achieved in ISIMIP3b-BASD, some artefacts are inherited from the coarser grid structure (e.g. higher precipitation over East Afghanistan). This shortcoming might influence the neighbour points at the edge of the artefacts with high precipitation differences. Previous studies have observed similar artefacts using the BASD algorithm as well^30,31^. The ISIMIP3b output for different variables has been evaluated globally, showing that they preserve the projected warming trend^43^. It has been shown that mean values are well-adjusted in most of the variables except for the snowfall flux^43^. To cross-check the validity of our product, we use available daily precipitation station observations in CA for the last 30 years in the data acquired from Meteostat (https://meteostat.net/en/). Only three observation sites were available for 1985-2000 (represented by squares in Fig. 3a), with fewer missing values after 2000 and at daily resolution. The right panels in Fig. 3 show the frequency of daily precipitation values between one original CMIP6 model (GFDL-ESM4-r1i1p1f1), its ISIMIP3b and ISIMIP3b-BASD products and the selected observation for each of the 3 cites. It can be seen that both BASD algorithms correct the bias of the original CMIP6 model, especially for smaller but more frequent daily precipitation values. Most of the precipitation amount in CA is accumulated over Kyrgyzstan and Tajikistan. The results for the other five models also show similar improvements (not shown).

## Results

### Internal model variability

**Figure 4:**
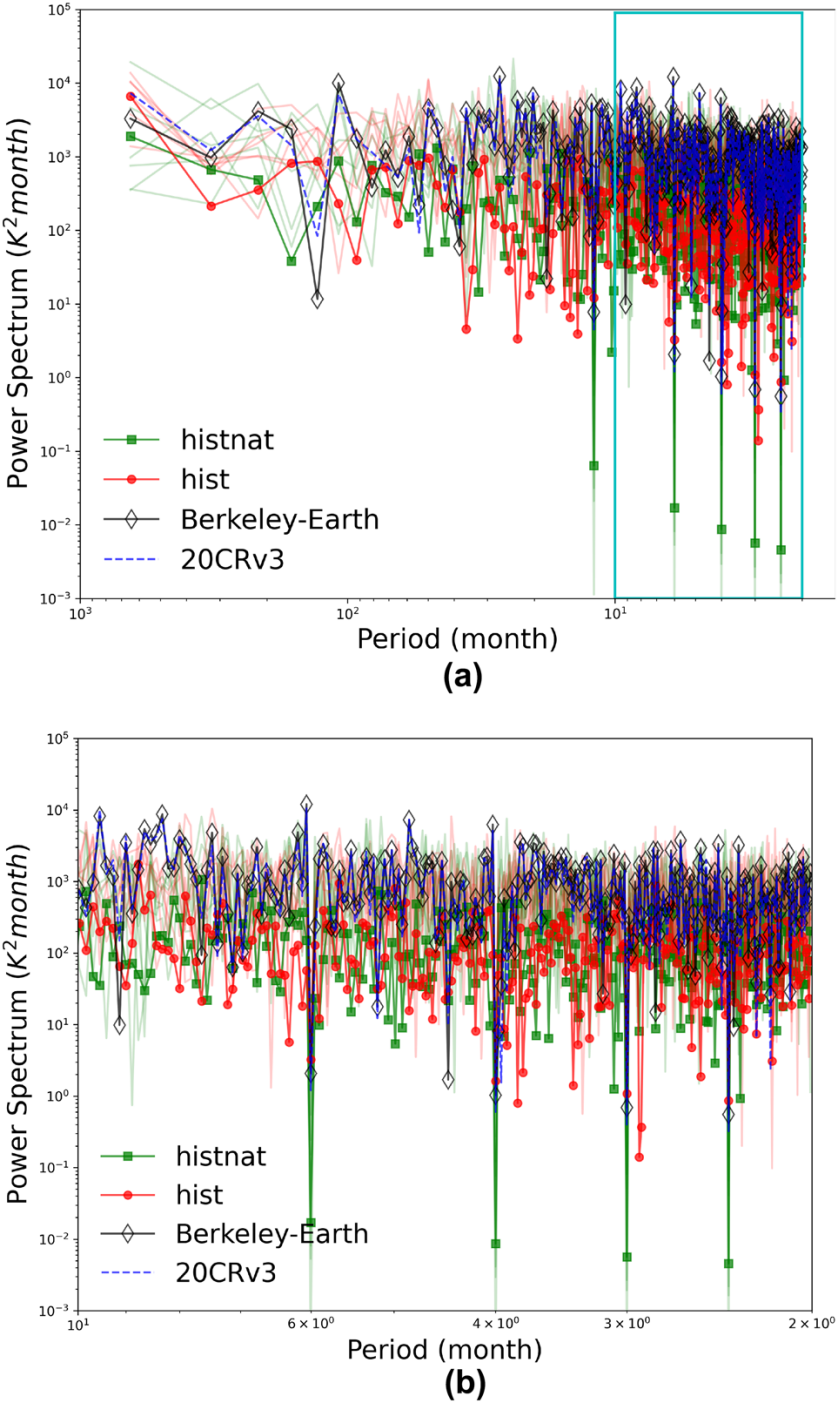
(**a**) Power spectra of monthly temperatures over CA (filed-mean) from *hist* (red), *hist-nat* (green), 20CRv3 (blue) and Berkeley-Earth (black) and (**b**) the zoom over the cyan box in (**a**).

Internal climate variability is usually referred to as unforced variability, which emerges from different components of the Earth system (i.e., atmospheric, oceanic, land, and cryospheric) and their coupled interactions^50^. Internal climate variability has an important impact on the projected climate at regional spatial scales and sub-decadal time steps^51^. Bias-adjustment methods usually do not discriminate between different sources of the model’s bias (uncertainty in scenarios and simulations or internal variability). Therefore, there is no guarantee that the internal variability remains preserved after the bias-adjustment^52^. It has been shown that the global mean temperature reproduced by climate models agrees with the observations in different frequency ranges. However, the energy differed by a factor of 2-3 for the 20-50-yr band between models^53^. Before any analysis, we investigate if the models’ variability is realistic compared to observations. Figure 4 compares the power spectrum of the detrended (the linear trend and the seasonal cycles are removed) monthly temperatures between the ensemble mean of *hist* and *hist-nat*, 20CRv3 and Berkeley-Earth. The power spectra of observations and reanalysis are a bit higher than the ones from the models. The differences in energy for higher frequencies are small. Both *hist* and *hist-nat* follow the observation and reanalysis well across all the frequencies. For precipitation, detecting the signal is usually challenging. Atmospheric circulations and internal variability impact precipitation trends more than radiative forcing. Therefore the uncertainty in the simulations is large, and the signal-to-noise (SNR) ratios are usually less than 2^51^.

**Figure 5:**
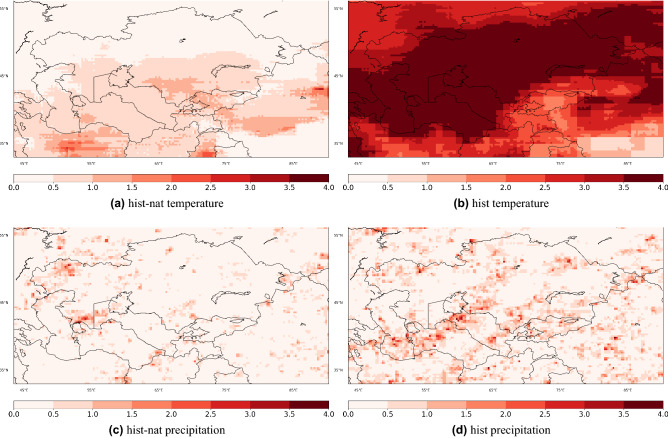
Signal-to-noise ratio maps for yearly temperature trends during 1979–2014 from *hist-nat* (**a**), *hist*(**b**), as well as for the yearly precipitation’s $99.9^{th}$ percentile from *hist-nat* (**c**), *hist* (**d**). The maps were created using python3-matplotlib (version 3.1.2, https://matplotlib.org/).

We calculate the SNR for the yearly temperature mean and yearly $99.9^{th}$ percentile of daily precipitation (Fig. 5). SNR is defined as the ensemble mean trend divided by their standard deviation over all the individual models^51^. PDF of the precipitation usually follows a gamma distribution, and for extreme value studies, the tail is of more importance. We explain the choice for selection of the $99.9^{th}$ threshold in Supplementary Information.

Figure 5 shows the SNR (unitless) maps for yearly temperature trends during 1979-2014 from *hist-nat* (a), *hist*(b), as well as yearly precipitation’s 99.9*th* percentile trends. It confirms the previous findings^51^ that the precipitation SNR remains almost less than 2 and that the model’s temperature’s SNR shows a stronger sensitivity due to the external forcing (Fig. 5b). The signal in the precipitation’s SNR in *hist* is slightly larger than the one from *hist-nat*. We will explore the precipitation signals in more detail in the following.

### Anthropogenic warming in CA

**Figure 6:**
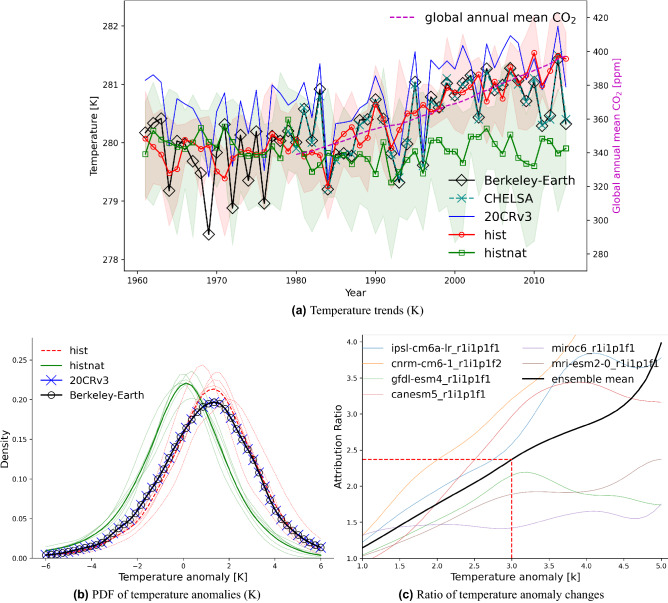
(**a**) Annual temperature time-series over Central Asia for the Berkeley-Earth data set^42^ (black line), historical models’ ensemble mean (red line), *hist-nat* models’ ensemble mean (green line) and cumulative global annual mean CO$_{2}$ concentration (ppm); (**b**) Normalized probability density functions of daily temperature anomalies (1995–2014 w.r.t. 1961–1980) for *hist* (solid red) and *hist-nat* (solid green), (**c**) ratio of daily temperature anomalies’ PDF (i.e., $\frac{P(tas_{hist})}{P(tas_{hist-nat}}$). Shadings in (**a**) show the ensembles’ spread.

Figure 6a shows the temperature time series of ISIMIP-BASD over CA for two simulation sets, *hist* and *hist-nat*, the Berkley-Earth and CHELSA observations and 20CRv3 reanalysis. The simulation driven by anthropogenic and natural forcing (*hist*) shows a constant positive trend similar to long-term observations from the Berkeley-Earth, CHELSA and 20CRv3 data sets (Fig. 6a). The simulations driven by natural forcing only (*hist-nat*) do not show any positive trend after 1979. The ensemble mean of the *hist* simulation exceeds the model spread of the *hist-nat* simulation after 2000. The cumulative global annual mean CO$_{2}$ exhibits an increasing trend similar to the observational temperatures after 1980. From 1980 to 2014, an increase of 60 ppm in global CO$_{2}$ concentration is accompanied by a warming of 1.5 K in the *hist* simulation over CA. The 20CRv3 reanalysis data shows a similar variability as in the Berkeley-Earth (Fig. 6b), however, with a warm bias. This shortcoming of the reanalysis has been previously shown in the average 500–1000-hPa layer temperature^54^.

**Figure 7:**
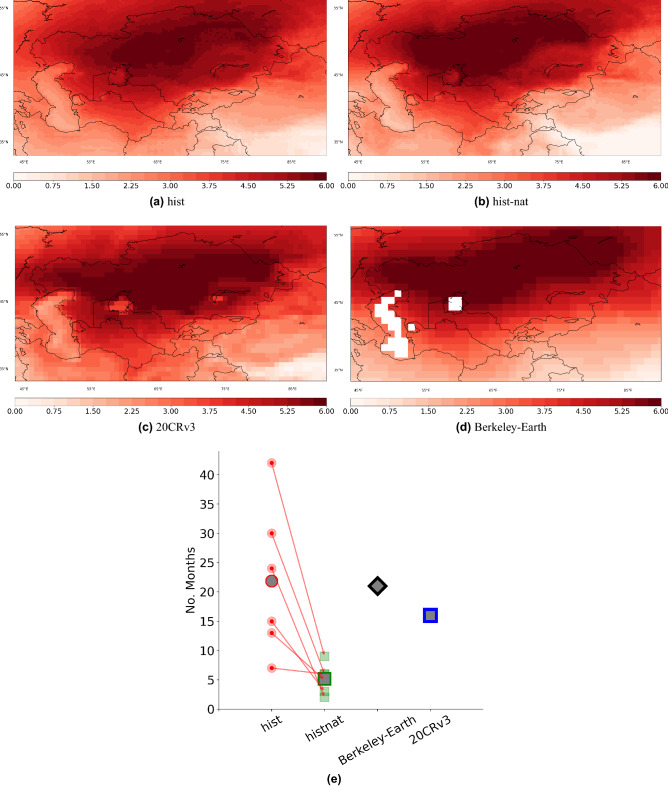
The mean temperature map for months with temperature anomaly greater than 3 K (1985–2014) from (**a**) *hist*, (**b**) *hist-nat*, (**c**) 20CRv3 and (**d**) same as in d but from the Berkeley observation data set. (**e**) shows the number of months detected with anomalies greater than 3 K for each data set. Ensemble mean of *hist* and *hist-nat* are shown by large circles and squares, respectively. The arrows connect the same models under different forcings. The maps were created using python3-matplotlib (version 3.1.2, https://matplotlib.org/).

On daily time scales, the normalized PDFs of temperature differences (averaged over CA) calculated between the mean values for the period 1995–2014 and 1961–1980, are significantly shifted towards warmer values by anthropogenic forcing (Fig. 6b). The PDFs of 20CRv3 and Berkeley-Earth are within the *hist*’s ensemble spread. The similarity of the PDFs of the *hist* ensemble average, 20CRv3 and Berkeley-Earth show that the ensemble simulation could realistically capture the temperature anomalies. In the next step, we use these PDFs to estimate the likelihood of temperature changes at different levels of warming. Dividing the likelihoods of *hist* by the one for *hist-nat* will indicate the attribution ratio of the anthropogenic forcing to each warming level (Fig. 6c). For example, a daily anomaly of +3K is more than two times more likely in an anthropogenic-forced climate across all six models considered. With regard to temperature anomalies larger than 3K, the ensemble spread of the attribution ratio becomes very large, and the different ensemble members fall into one of two branches. Three models (IPSL-CM6A-LR, CNRM-CM6-1 and CANESM5) show an exacerbating trend in the attribution ratio, while the remaining three (MIROC6, MRI-ESM2-0 and GFDL-ESM4) a flat one. To explore spatial patterns corresponding to the +3 K temperature anomaly, we calculate the ensemble mean of temperature anomalies for months with regionally averaged values (over CA) of at least 3 K warmer than the climatology (1961–1980) from the ensemble of the *hist* and *hist-nat* simulations in Fig. 7. In all datasets, the resulting pattern reveals more than 5K warming over Kazakhstan and lower warming over the rest of CA. The three models having a flat trend in attribution ratio (MIROC6, MRI-ESM2-0 and GFDL-ESM4) show a higher warming level in Kazakhstan than the remaining models (not shown here). The overall warming pattern, however, is similar to the ensemble mean. To explore if such a warming pattern exists in the real world, we calculate the same composites as in Fig. 7c,d from 20CRv3 reanalysis and Berkeley-Earth data, i.e. an average of months with regionally averaged values (over CA) of at least 3 K warmer than the climatology (1985–2014 w.r.t. 1961–1980). Number of months with temperature anomalies larger than 3 K in Berkeley-Earth and the ensemble mean of *hist* are similar, and *hist-nat* shows a significantly smaller number (Fig. 7e). The similarity of extreme temperature patterns in the Berkeley-Earth, 20CRv3 data sets and +3K warming level patterns of the *hist* and *hist-nat* simulations demonstrate that observed and simulated warming for the area has a similar pattern. Given the shift in PDF of temperature anomalies shown in Fig. 6b in *hist*, the probability of such wide-spread warming events is shown to increase compared to *hist-nat* at least by factor 2.5 (Fig. 6c). We conclude that the sensitivity of the simulated temperature for *hist* to anthropogenic forcing is significantly high.

### Anthropogenically forced precipitation

**Figure 8:**
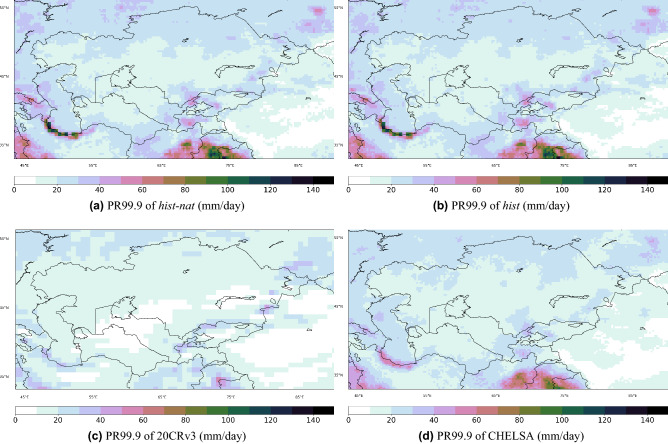
Extreme precipitation patterns [mm/day] calculate as 99.9$^{th}$ percentile of daily values for the overlapping period of 1979–2014 for (**a**) CHELSA, (**b**) models’ ensemble mean of the historical run. All the datasets are at 0.25$^{\circ }$ resolution. The maps were created using python3-matplotlib (version 3.1.2, https://matplotlib.org/).

**Figure 9:**
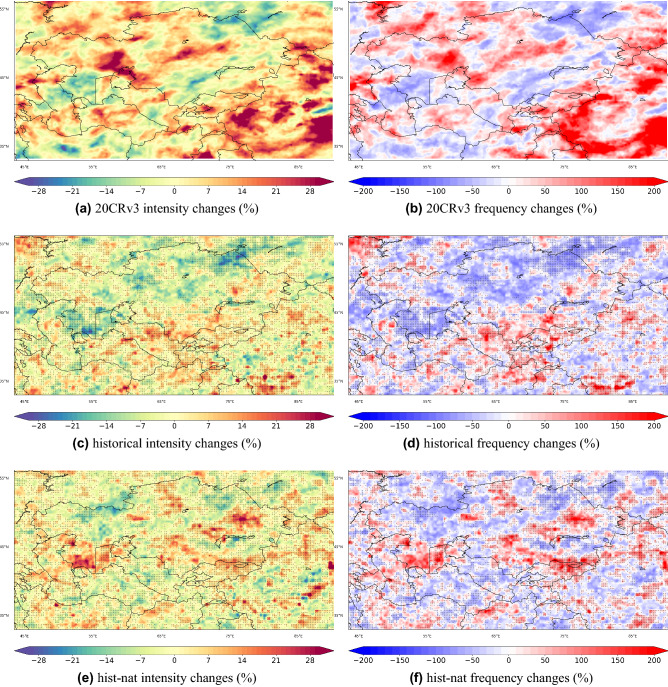
Percent of changes (1995–2014 vs 1961–1980) in the intensity and frequency of 99.9$^{th}$ total daily precipitation percentile for 20CRv3 (**a**,**b**); *historical* (c,d) and *hist-nat* (**e**,**f**), respectively. The 99.9$^{th}$ happens on average once in 1300 days in the 20CRv3 data. Black dots indicate the agreement in the signs with 20CRv3. The maps were created using python3-matplotlib (version 3.1.2, https://matplotlib.org/).

For detecting heavy precipitation events, we consider values larger than 99.9 percentile of daily precipitation (PR99.9), which is a common threshold for detecting rare events^10^ (other percentiles, i.e. 98, 99 and 99.7 are discussed in Supplementary Materials). PR99.9 from CHELSA, 20CRv3, *hist* and *hist-nat* ensemble means for the overlapping period of 1979-2014 are shown in Fig. 8. The overall patterns are similar with an overestimation of values in *hist* and *hist-nat* over the North and West of Iran, Afghanistan, and North India^43^. Due to the lower resolution, the precipitation values over mountainous regions are lower in 20CRv3. We will focus on the changes in the intensity presented in Eq. (1) and frequency in Eq. (2) of extreme precipitation events, where *N* in Eq. (2) indicates the number of events at each grid point of the domain which is greater or equal to *PR*99.9 for the selected time period. Figure 9 shows the percentage of changes in intensity and frequency of PR99.9 in *hist*, *hist-nat* and 20CRv3.

1 $$\begin{aligned}{} & {} \frac{PR99.9_{1995-2014} - PR99.9_{1961-1980}}{PR99.9_{1961-1980}} \times 100 \end{aligned}$$

2 $$\begin{aligned}{} & {} \quad \frac{N_{(PR_{1995-2014}> PR99.9)} - N_{(PR_{1961-1980}> PR99.9)}}{N_{(PR_{1961-1980} > PR99.9)}} \times 100 \end{aligned}$$

**Figure 10:**
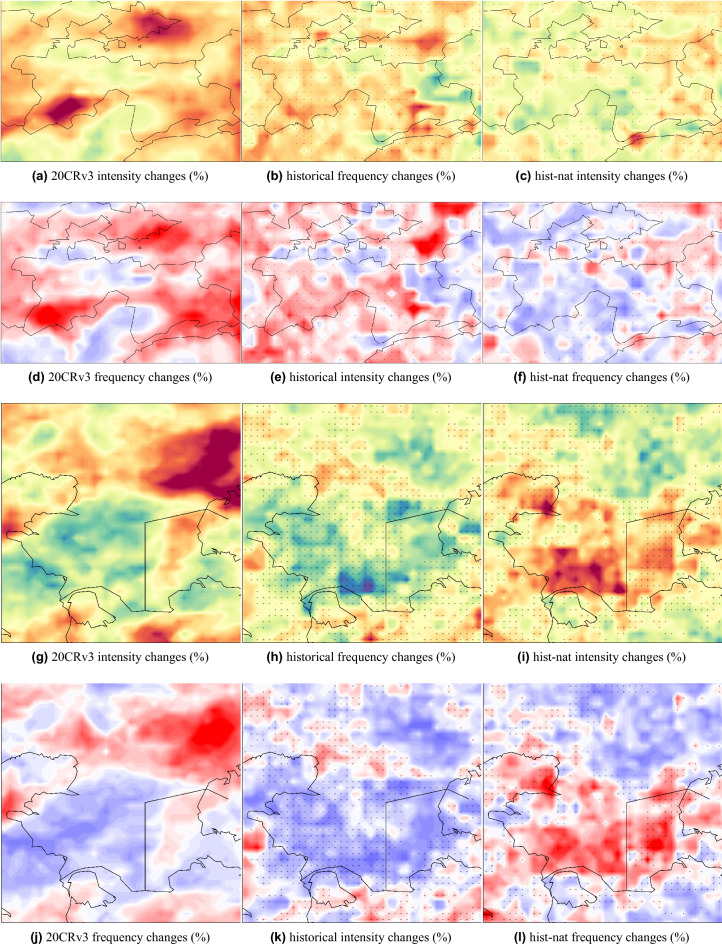
As in Fig. 9 but the zoom of all maps over Tajikistan is shown in (**a**)–(**f**) and over Mangystau Region in Southwest Kazakhstan (**g**)–(**l**). The maps were created using python3-matplotlib (version 3.1.2, https://matplotlib.org/).

**Figure 11:**
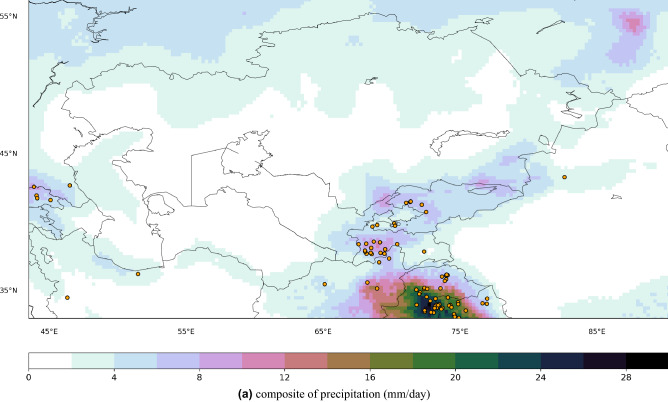
Composite of precipitation during the rainfall-triggered landslide events [mm/day] from CHELSA, (**b**) changes in models’ ensemble mean of the number of days with precipitations greater than 20 mm (eca$\_$r20mm) in the 1979–2014 period, i.e. *hist* minus *hist-nat*. Orange dots indicate the rainfall-triggered landslide locations from GFLD and GLC datasets. The maps were created using python3-matplotlib (version 3.1.2, https://matplotlib.org/).

Overall, areas with an increased/decreased intensity coincide with areas of increased/decreased frequency. An intensifying and increased frequency of PR99.9 pattern is observed over the Tibetan Plateau, West China, Ahal in Central Turkmenistan, North and northwest Kazakhstan, Khatlon in South Tajikistan, Ferghana in South Kyrgyzstan, Southeast Afghanistan, and Northwest Iran. The intensity and frequency of extreme precipitation events are decreased over the Mangystau Region, Jambyl, Turkistan, Pavlodar and Akmola in Kazakhstan, Central Iran, and East Turkmenistan. Such patterns can intensify at each level of projected warming^6^. The ensemble mean of *hist* simulations could capture some of the increasing and decreasing patterns seen in observations and not represented by *hist-nat*. For example, the simulated decrease in intensity and frequency over the Mangystau Region in West Kazakhstan and the Central Caspian Sea agrees well with the 20CRv3 (Fig. 10g–l). The increase in the intensity and frequency over the Khatlon area in Southwest Tajikistan is captured only by the *hist* simulation, while *hist-nat* shows a decreasing signal (Fig. 10a–f). This area has a potential for rainfall-triggered landslide events^23^. To explore the patterns of precipitation associated with the landslide events, we plot the composite map of precipitation from CHELSA during the selected landslide events. The resulting map will show the average precipitation when those landslide events occur in the domain. Figure 11 reveals a pattern similar to the one in PR99.9 in Fig. 8. The orange dots in Fig. 11 show the locations of the rainfall-triggered landslide events in CA in 2004–2014. As shown over the mountainous areas of Tajikistan and Kyrgyzstan, the landslide events fall within the areas with anthropogenic-enhanced PR99.9 values. This shows the associated pattern of extreme precipitation events and emphasizes the risk of increasing extreme precipitation events, which were captured only by the *hist* and 20CRv3 over the mountainous areas of Tajikistan and Kyrgyzstan (Fig. 10a–f). The *hist-nat* ensemble mean shows a reduction in intensity and frequency of RP9.9 over this area (Fig. 10c,f). The presented maps agree well with the previous findings^23^ showing an increase in the total annual wet-day precipitation, daily intensity index, extreme wet days (RP95) and maximum 5-day precipitation amount from 15 CMIP5 GCMs during 1961-2005. The sensitivity of the *hist* precipitation to the anthropogenic forcing does not present a large scale pattern as seen in the temperature, and only regional signals have been detected (Figs. 9 and 10). Neither the patterns of precipitation’s intensity and frequency changes show the same similarity with the reanalysis as seen in the temperature signals (Fig. 7). Therefore the presented data set might produce a larger anthropogenic influence in the impact studies, which are influenced more by the temperature than by the precipitation. We propose to examine the influence of our climate input data using an impact model to shed light on the role of local precipitation intensity and frequency changes.

## Discussion

Regional impact studies require bias-adjusted and downscaled climate data. However, one has to evaluate such products over the region of interest and know their limitations and shortcomings. Impact studies focusing on the anthropogenic influence sometimes require a so-called “counterfactual”^55^ baseline climate data which accounts for the human-induced influences. In this study, we downscale two scenarios of the ISIMIP3b data set, with and without anthropogenic forcing, to analyze the impact of human-induced warming on the intensity and frequency of extreme temperature and precipitation events over CA. The regional temperature increase in CA is higher than the global warming level^56^. We observed accelerated regional warming, which eventually leads to significant changes in precipitation patterns over time and space, confirming previous studies^57^. It is known that an increase in the surface sensible heat over the Central and Eastern Tibetan Plateau intensifies the summer drought in arid areas of CA and weakens the “South Asia High”, and subtropical jet stream^58^. It has been previously shown that increased greenhouse gases in the atmosphere move the subtropical westerly jet southward, causing a deficit in the hydrological cycle of the area^59^. However, warmer and wetter climates are already replacing the cold climate in mountainous areas of CA^60^. Moreover, extra water vapour from the western Pacific, North Atlantic and Arctic can converge in CA and contribute to the extreme precipitation events^58^.

Our analysis shows an increased risk of extreme warm events under anthropogenic forcing, especially in Kazakhstan, with possibly devastating consequences for the area’s population. This agrees with the observations showing a temperature increase of at least 5K in some regions of Kazakhstan between 1990 and 2020, which are now classified as “Temperate-cold deserts”. Since the 1980s, a large part of CA characterized by desert climate has expanded about 100 kilometres northward in southern Kazakhstan, northern Kyrgyzstan, and Uzbekistan^60^. It has been shown previously that a 3K warming level in CA will increase the risk of mud-flow by tenfold in Kazakhstan^61^. We find a significant local enhancement of heavy precipitation over the mountainous area of CA, e.g., Kyrgyzstan and Tajikistan, known as the “water reservoirs” for the arid and semi-arid CA, susceptible to the effect of anthropogenic forcing. Severe precipitation and temperature patterns derived from the downscaled *hist* model simulations show similar patterns as in the reanalysis and observation data sets. A significant rise in the frequency and magnitude of extreme events (floods, droughts and heatwaves) in CA has been reported for recent decades, primarily based on precipitation and temperature changes^7,62^. Here, we could attribute some of those changes to anthropogenic forcing and exhibit the associated extreme precipitation and temperature patterns. Therefore, the downscaled data can potentially implement anthropogenic influences for future impact studies and can be used as climate input data in the impact models. We conclude that the community could use our presented framework/dataset for detecting and attributing extreme events in CA or elsewhere. The *hist-nat* simulation could efficiently remove climate change signal from *hist*. We found a correlation between extreme precipitation events and landslide events. In a simulation without anthropogenic forcing, extreme precipitation events’ frequency and intensity decreased significantly over the mountainous area of CA compared to the full-forced simulations. Given that the anthropogenic forcing had reached higher levels after 2014 (unprecedented CO$_{2}$ concentrations), such extremes can increase in magnitude and number in the future. Possible applications of our data set can be to study the anthropogenic impacts on the glacial and snow melt, river flow, agriculture, food security, human health, energy and migration.

Several caveats of our analysis might be as follows. Due to computational constraints, we could not downscale a more extensive set of simulations that could better sample the extreme value distributions like the PDF of precipitation events or decrease the sampling uncertainty. For *hist* and *hist-nat* experiments, using one run per model is inappropriate, and we might underestimate the uncertainty. Long-term change attribution or event attribution requires as many runs as possible when using CMIP6-like simulation, often more than three runs per model^15^. If using a single member per model for event attribution, one needs to use a larger sample size (sample size = $N_{models}\times N_{realizations}\times N_{years}$)^15^. The sample size of ISIMIP might not meet those requirements. Usually, the CMIP6 or downscaled CMIP6 model used for both long-term change attribution and event attribution must choose multiple runs in multiple models^63^. However, we provided and tested a methodology which other researchers could use to increase the sample size presented here in the future. We justify our selection of the 6 ISIMIP models because the impact models are usually tuned against the observations and therefore require bias-adjusted and downscaled climate data. ISIMIP provides climate information using a limited number of CMIP6 GCMs, which span the range of global mean temperature change ($\Delta$GMT) and relative precipitation changes^64^. On the other hand, in most bias-adjustment methods (including this study), there is no guarantee that the physical consistency remains preserved among the model variables. The ISIMIP-selected models build a counterfactual baseline climate state for impact studies. We have shown that the *hist-nat* ensemble mean could remove the observed warming trend. Therefore, we conclude that our methodology would be an alternative to simple detrending studies^55^, which produce such baseline climate states. At the moment, due to the computational limitations, it is impossible to use our bias adjustment and statistical downscaling across many members of CMIP6 models. The CHELSA data set is based on the observational network, which is sparse for the daily precipitation values over CA, especially in the years after the dissolution of the Soviet Union. Landslides might be linked to heavy precipitation on previous days. Although we have chosen a three-day running window, in some cases, the precipitation period might be prolonged to weeks before the event. Even moderate rainfall after a long drought (“soil sealing”) might also lead to landslides which we did not consider in the analysis. The number of rainfall-triggered events is also limited since such data sets are not freely available in CA countries. Despite the efforts to collect extreme climate records like the https://floodlist.com/, https://www.emdat.be and https://global-flood-database.cloudtostreet.ai/ (last access on 7 July 2022), a comprehensive extreme event database is missing in CA. For example global flood database reports insufficient flood exposure data coverage in Turkmenistan, Tajikistan and Kyrgyzstan.

Finally, it should be noted that the need and demand for climate information can differ greatly from the availability. As climate scientists, we always have to deal with compromises and considerations because we are at the very beginning of the chain of climate impact research. It is, therefore, an important task for us to distort the nature of the changes and to soften existing rules with care. Reducing model ensembles is an essential aspect, which becomes more important with the increasing refinement of the models since the resources are also limited in the digital world.

## Supplementary Information

Supplementary Information.

## Data Availability

The downscaling datasets generated during the current study are available freely at https://zenodo.org/record/7063876#.YxsOF1xByhd. Other datasets used are introduced in the Data and Methods section. The ISIMIP3 BASD code (version 3.0.1) is freely available at https://zenodo.org/record/6758997. The ISIMIP3b data can be accessed via https://data.isimip.org/. The GCMs were selected due to the availability of natural and anthropogenic plus natural forced experiments from the ISIMIP3b project. The selected models are IPSL-CM6A-LR-r1i1p1f1^65^, CNRM-CM6-1-r1i1p1f2^66^, GFDL-ESM4-r1i1p1f1^67^, CANESM5-r1i1p1f1^68^, MIROC6-r1i1p1f1 (https://www.wdc-climate.de/WDCC/CMIP5/Compact.jsp?acronym=MIM5r8; last access 6.07.2022) and MRI-ESM2-0-r1i1p1f1^69^. The aggregated CHELSA data sets are publicly available via https://data.isimip.org/10.48364/ISIMIP.836809.2. The Global Landslide Catalog can be found at https://data.nasa.gov/Earth-Science/Global-Landslide-Catalog-Not-updated-/h9d8-neg4. The Global Fatal Landslide Database is freely available at https://svs.gsfc.nasa.gov/4710. The open-access land-slide data are usually based on citizen science and media reports with a layer of expert review. Some of the most remarkable events in terms of the impact on the population are the Murghab landslide in Tajikistan, with 20 fatalities, triggered by heavy rain on the 21st of July 2007, the Abi Barik Village landslide in Badakhshan, Afghanistan, with 2100 deaths, triggered by continuous rainfall occurred on the 2nd of May 2014 and the Gorno-Badakshan area land-slide in Tajikistan with 20 deaths and triggered by rainfall which happened on the 21st of July 2007. The global annual mean CO$_{2}$ data are downloaded via URL: gml.noaa.gov/. The Berkeley-Earth data set^42^ is available freely at http://berkeleyearth.org/data/. The NOAA-CIRES-DOE 20th Century Reanalysis version 3 ensemble mean (20CRv3) daily precipitation data can be downloaded from https://psl.noaa.gov/data/gridded/data.20thC_ReanV3.html. For manipulation of the netcdf files, we used CDO (version 2.0.3; https://mpimet.mpg.de/cdo) and NCO (version 5.0.6 ). For data analysis and plotting, we used the jupyterhub at DKRZ and the following python3.9.9 packages: basemap, dask, matplotlib, numpy, rasterio, earthpy, IPython and joblib.
