# Supplementary material for: Anthropogenic influence on extreme temperature and precipitation in Central Asia
Source: Sci Rep. 2023 Apr 26;13:6854. doi: 10.1038/s41598-023-33921-6 (PMC10133278; doi:10.1038/s41598-023-33921-6)
Supplement: Supplementary file 1 — Supplementary Information. [file 41598_2023_33921_MOESM1_ESM.pdf]

Supplementary Information for “Anthropogenic influence on extreme temperature and precipitation in Central Asia”[1]

Here we show the percent of changes (1995-2014 vs 1961-1980) in the intensity and frequency of different percentiles of total daily precipitation percentiles for 20CRv3; historical and histnat, respectively. Black dots indicate the agreement in the signs with 20CRv3. The maps were created using python3-matplotlib (version 3.1.2, https://matplotlib.org/).

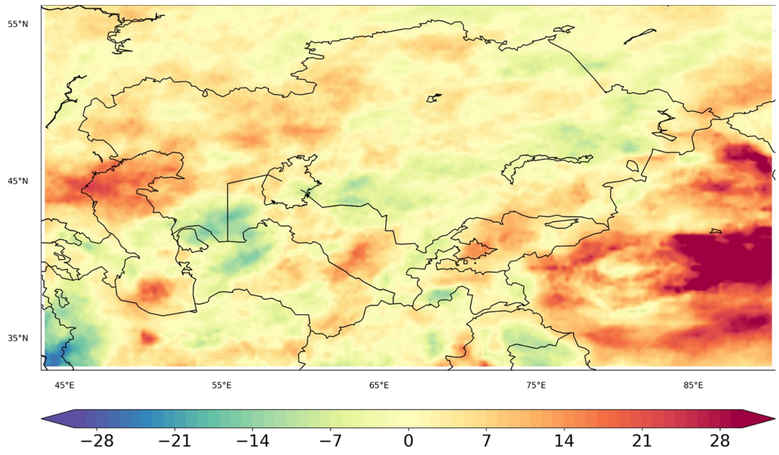

S1. 98th 20CRv3 intensity changes

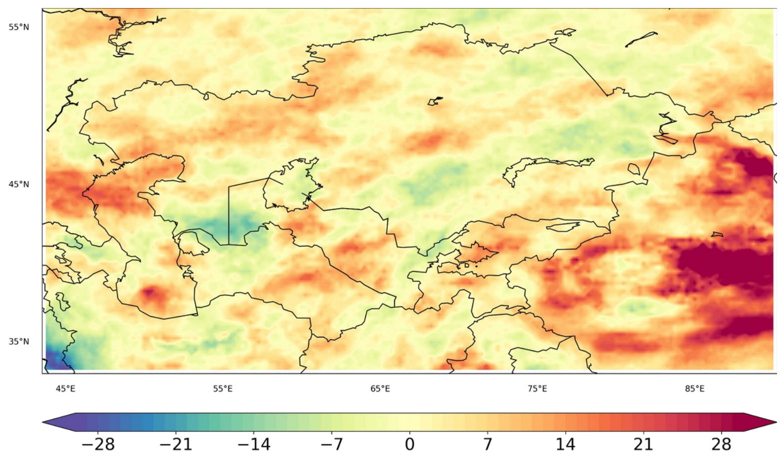

S2. 99th 20CRv3 intensity changes

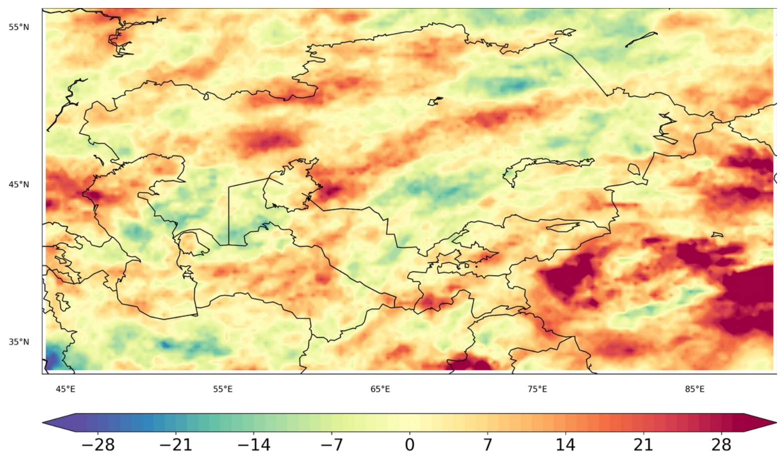

S3. 99.7th 20CRv3 intensity changes

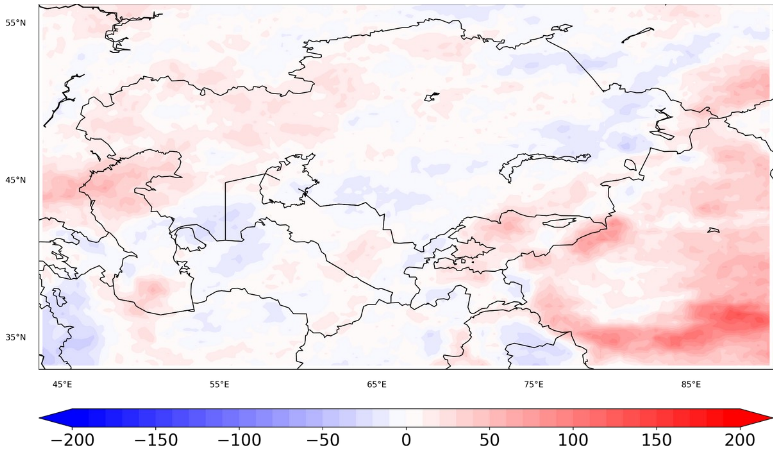

S.4 98th 20CRv3 frequency changes

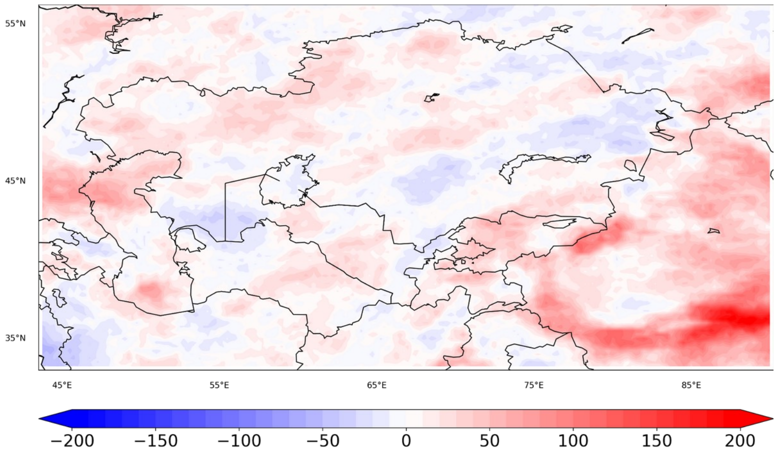

S.5 99th 20CRv3 frequency changes

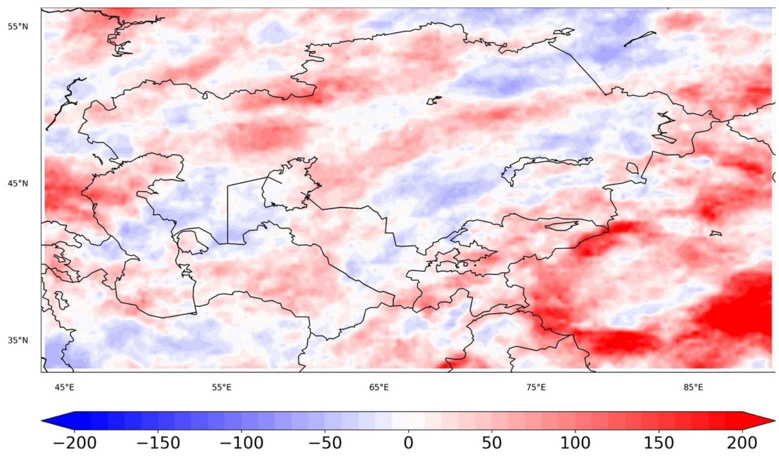

S.6 99.7th 20CRv3 frequency changes

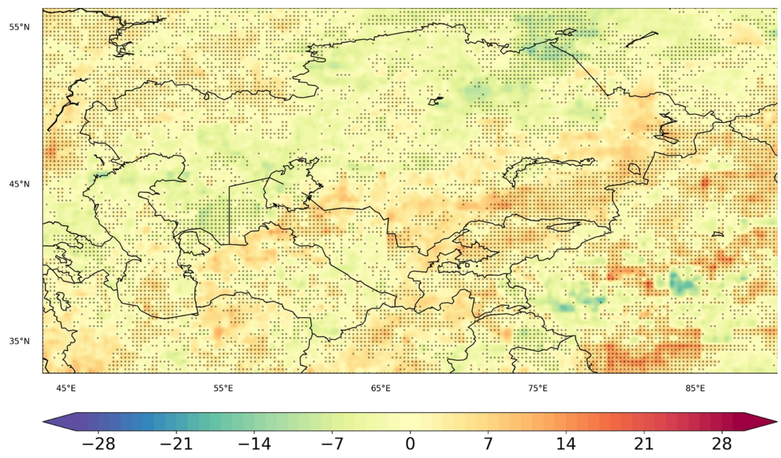

S.7 98th historical intensity changes

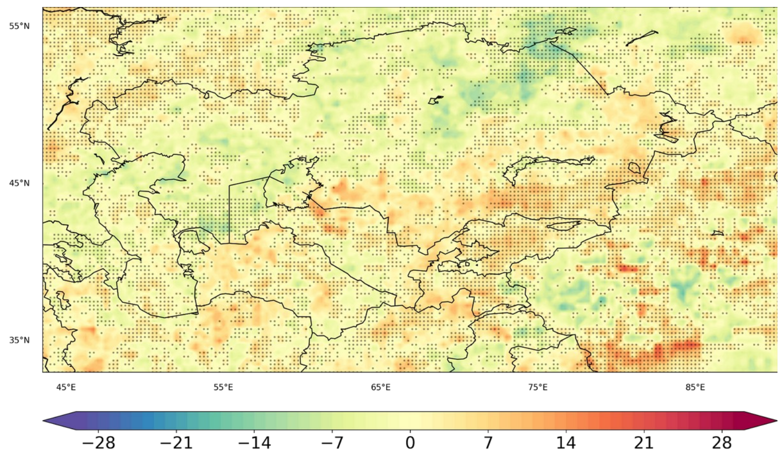

S.8 99th historical intensity changes

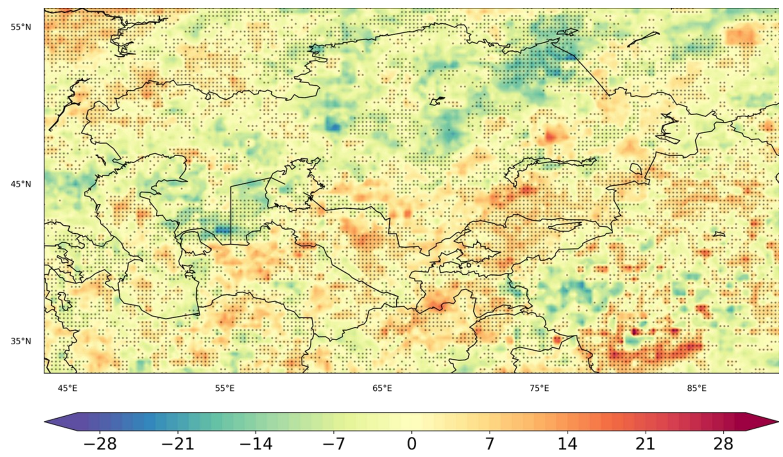

S.9 99.7th historical intensity changes

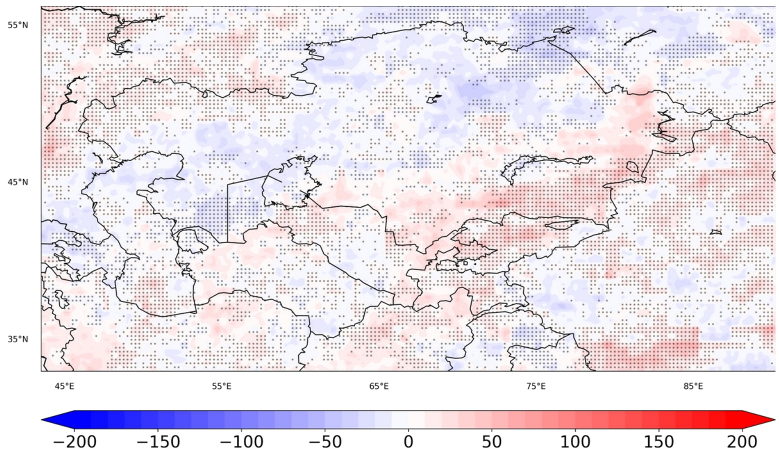

S.10 98th historical frequency changes

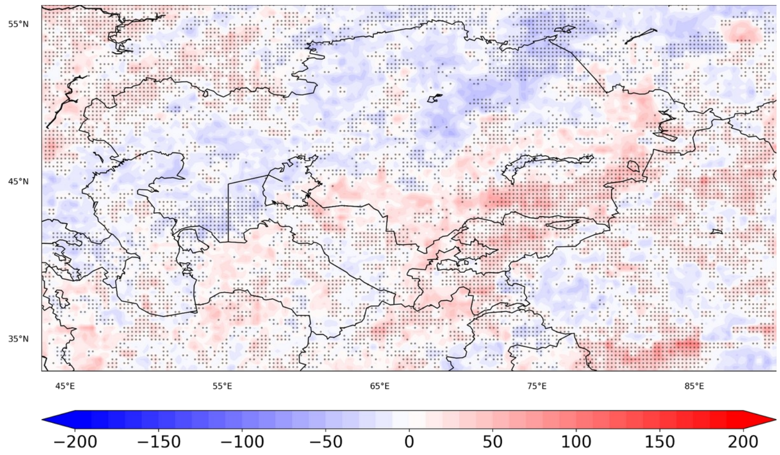

S.11 99th historical frequency changes

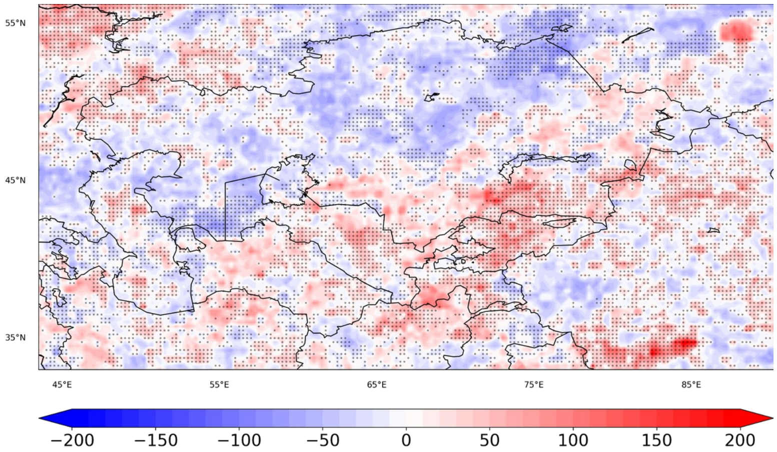

S.12 99.7th historical frequency changes

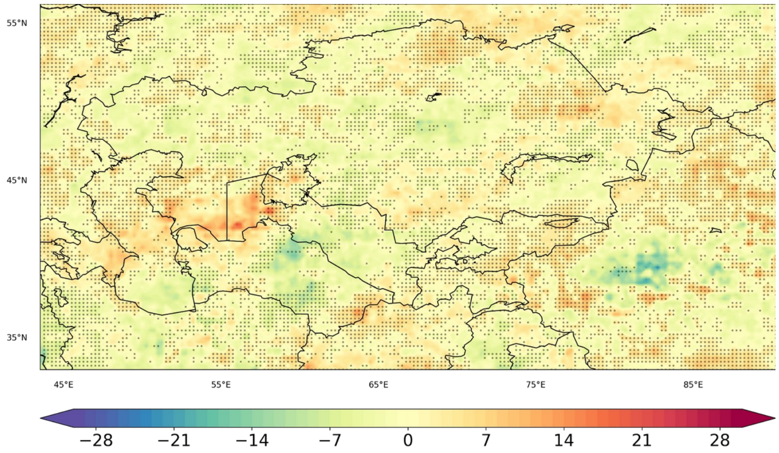

S.13 98th histnat intensity changes

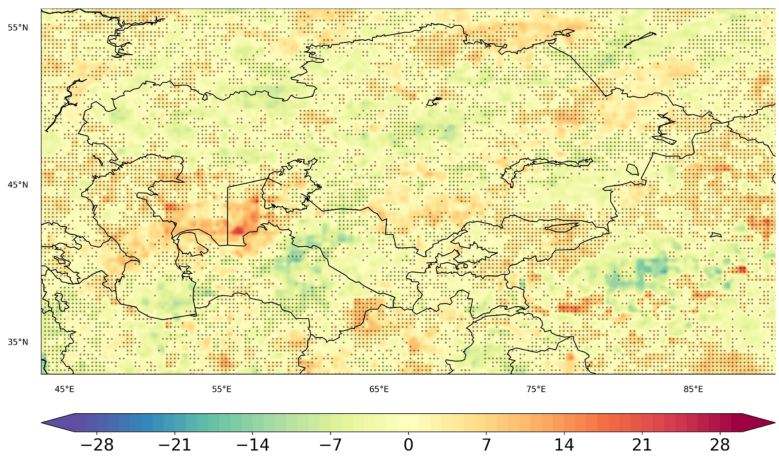

S.14 99th histnat intensity changes

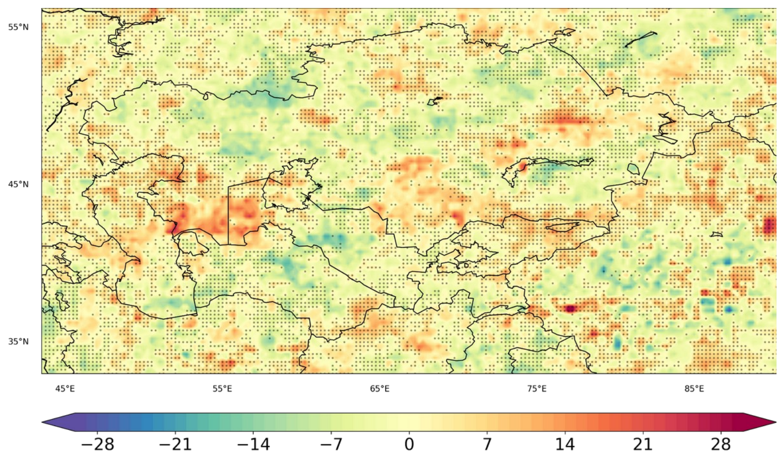

S.15 99.7th histnat intensity changes

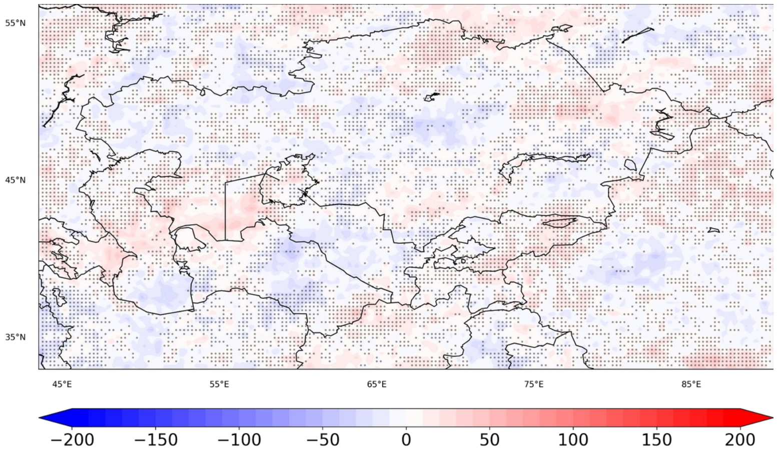

S.16 98th histnat frequency changes

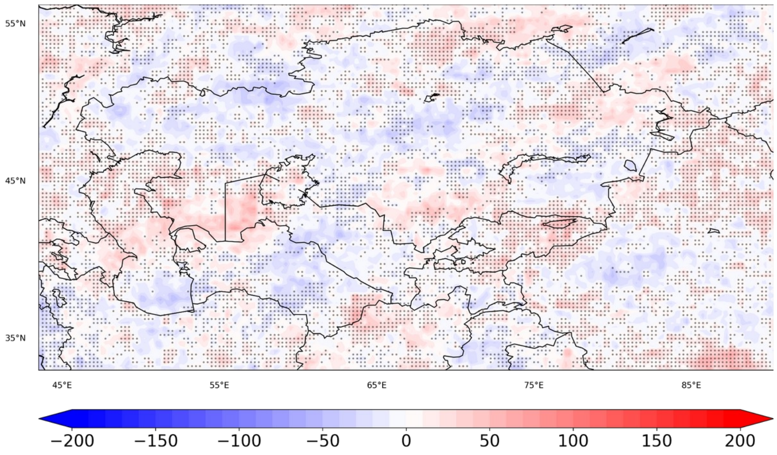

S.17 99th histnat frequency changes

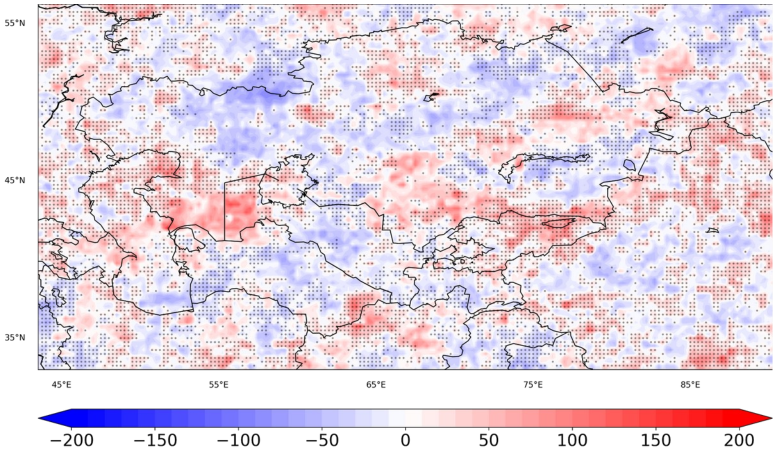

S.18 99.7th histnat frequency changes

We have chosen a region over the landslide locations in Tajikistan to explore the increase in the precipitation PDF. The blue box in Figure S.19 shows the region selected. The differences between the PDFs of hist and histnat are shown in Figure S.21. As can be seen, the probability of more extreme precipitations is higher in the hist compared to histnat.

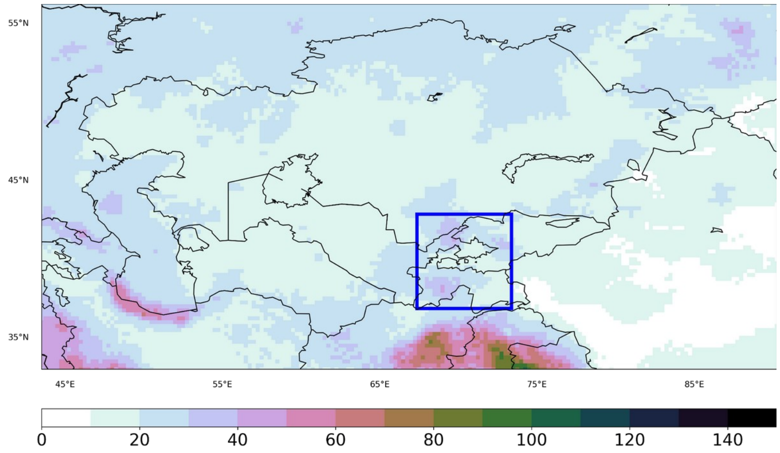

S.19 PR99.9 from CHELSA

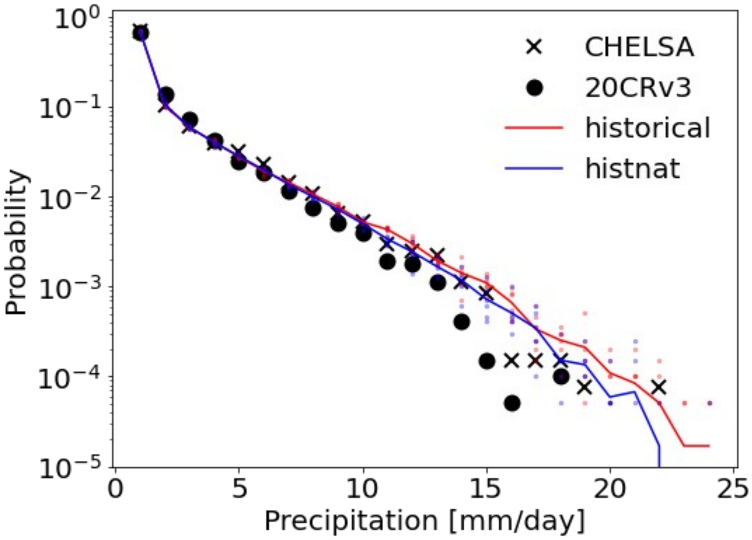

S.20 PDF of field mean precipitation over the blue box of figure S.19. Solid lines show the ensemble mean.

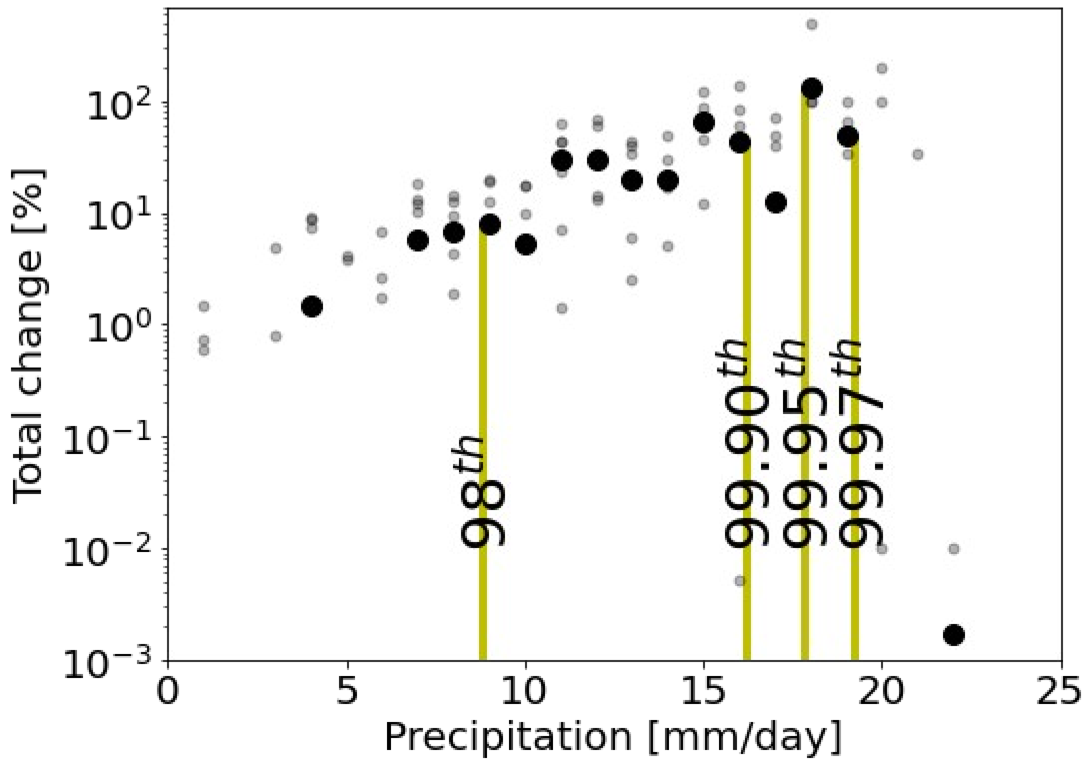

S.21 Total changes of PDF [%] between hist and histnat. Black solid circles show the ensemble mean and smaller ones individual models.
